# Effects of Exogenous Dopamine on the Uptake, Transport, and Resorption of Apple Ionome Under Moderate Drought

**DOI:** 10.3389/fpls.2018.00755

**Published:** 2018-06-05

**Authors:** Bowen Liang, Tengteng Gao, Qi Zhao, Changqing Ma, Qi Chen, Zhiwei Wei, Cuiying Li, Chao Li, Fengwang Ma

**Affiliations:** State Key Laboratory of Crop Stress Biology for Arid Areas, Shaanxi Key Laboratory of Apple, College of Horticulture, Northwest A&F University, Yangling, China

**Keywords:** apple, dopamine, water stress, mineral nutrients, leaf senescence

## Abstract

The frequency and intensity of water deficits is expected to increase because of global warming. Drought stress is often one of the most limiting factors for plant growth. We conducted greenhouse pot experiments to address how dopamine affects the drought-resistance traits of apple trees at the physiological and molecular levels. Our factorial design consisted of dopamine and no-dopamine applications combined with well-watered and moderate-drought conditions. Seedling biomass, photosynthesis rates, chlorophyll concentrations, and stomatal apertures were markedly reduced under stress but dopamine treatment mitigated the inhibiting effects of drought on plant growth and helped maintain strong photosynthesis, chlorophyll levels, and stomatal functioning. Concentrations of most macro-, micro-, and trace elements decreased in response to drought. This stress also diminished the uptake and transport of elements in the leaves and stems, but increased the partitioning of elements in the roots. Nutrient resorption proficiency decreased while nutrient resorption efficiency increased for most analyzed elements. Exogenous dopamine significantly increased the concentrations, uptake, and transport of nutrients under drought stress, and also altered their distribution within the whole plant. However, this molecule had a negative effect on nutrient resorption. Although transcript levels of a key chlorophyll degradation gene, *pheide a oxygenase*, and *senescence-associate gene 12* were elevated upon drought treatment, dopamine significantly suppressed the upregulation of those genes under such stress conditions. These observations indicate that dopamine has an important anti-senescence effect that might be helpful for regulating nutrient uptake, transport, and resorption, and ultimately influencing overall plant growth. Thus, understanding the role of dopamine in drought tolerance introduces new possibilities to use this compound for agricultural purposes.

## Introduction

In arid and semi-arid regions, water is one of the most limiting factors that influence many morphological, physiological, biochemical, metabolic, transcriptomic, and proteomic processes, thereby affecting plant growth, production, and survival (Tomlinson et al., 2012; Alam et al., 2017). Among the typical abiotic stresses, drought is globally the most devastating to growth and productivity (Ryan, 2011). Over the past few decades, the frequency and intensity of regional and global extremes in water deficits have increased. Its impact on growth, morphology, and physiological processes within the aboveground and belowground parts of a plant can be measured in several ways, e.g., gas exchange, stomatal conductance, water relations, root longevity, leaf water potential, activity of phytohormones and reactive oxygen species, cell division, nutrient assimilation and transport, and metabolic processes (Ergo et al., 2018; Rahmati et al., 2018). Under drought conditions, plants generally close their stomata to minimize water losses, thereby reducing photosynthetic capacity. The combined effects of restricted CO_2_ uptake, decreased photosynthesis, less cell expansion, and changes in nutrient status cause growth to slow or cease, thus diminishing biomass production (Tschaplinski et al., 1998; Verslues et al., 2006). Although drought stress can limit yields and plant survival, adaptations can be made to plant physiology, morphology, and metabolism as well as to other complex mechanisms to bring a balance between water lost through transpiration and water taken up by the root system (Thapa et al., 2011). Because plants avoid or tolerate drought in many ways, ranging from signal perception and transduction to regulation of gene expression and metabolic changes, research that focuses on only one of those components will probably not produce conclusive results (Chaves et al., 2003).

Mineral nutrition plays an important role in plant growth, development, productivity, and water relations (Goldstein et al., 2013). Long-distance water transport in plants can be modulated by altering the concentration of cations in the xylem sap (Nardini et al., 2011). This phenomenon, the ‘ionic effect,’ is likely due to ion-mediated volume changes in the pectins found in pit membranes and/or electroviscous results in pit apertures (Lee et al., 2012; Santiago et al., 2013). The ionic effect also has a valuable role in optimizing the delivery of water and nutrients to different plant sectors and in regulating resistance to drought stress (Sellin et al., 2010; Oddo et al., 2011). Many experiments have demonstrated that adequate mineral nutrition is fundamental to hydraulic properties. Mass flow, diffusion, and contact exchange are the three major mechanisms for mineral element uptake in higher plants. The diffusion coefficient strongly depends upon soil water potential, decreasing when that potential reduces. Thus, nutrient uptake is highly affected by soil water potential and is restricted under drought conditions (Salehi et al., 2016). Deficient levels of minerals lead to growth inhibition and yield loss because of their physiological and biochemical functions. For example, reduced availability of nitrogen and potassium affects xylem hydraulic capacity and increases vulnerability to cavitation due to structural modifications of the xylem conduits (Harvey and van den Driessche, 1999; Hacke et al., 2010). Furthermore, potassium is critical to many processes related to drought adaptations, and maintaining an optimal level of that element can reduce the effects of such stress (Grzebisz et al., 2013; Wang and Wu, 2017). Water deficits can influence plant nutrient status in three ways. First, the soil moisture content and soil nutrient mineralization are reduced, which decreases the available pools of nutrients in the soil (Sanaullah et al., 2012). Second, less mass flow or diffusivity inhibits nutrient uptake and transport (Fageria et al., 2002). Third, photosynthesis and transpiration rates are altered due to reduced stomatal conductance and assimilation (He and Dijkstra, 2014). Although previous investigations of drought stress have focused on water relations, gas exchange, assimilation, and growth, little information has been reported about the effects of drought on nutritional status.

Catecholamines are a group of biogenic amines with a 3, 4-dihydroxy-substituted phenyl ring. They include dopamine, norepinephrine, epinephrine, and their derivatives. Dopamine, a natural product of the catecholamine pathway, is a well-known neurotransmitter in mammals (Wang et al., 2018). In contrast to the vast amount of knowledge about its the role and effects in mammals, little is known about the physiological significance of dopamine in plants. This water-soluble molecule was first identified in plants as having strong anti-oxidative capability that was greater than glutathione, catechin, the flavonol quercetin, and the flavone luteolin, and similar to that of gallocatechin gallate and ascorbic acid (Kulma and Szopa, 2007). Dopamine influences sugar metabolism and coordinates with phytohormones to affect plant growth (Jung et al., 2000). It can accelerate cell expansion on a growth medium supplemented with indoleacetic acid and kinetin but is useless for cells incubated on a basal medium (Protacio et al., 1992). Dopamine plays an important role in the intercellular regulation of ion permeability and photophosphorylation of chloroplasts due to its reduction power that ends with the scavenging of free radicals (Roshchina, 1990). It enables organisms to fine-tune their stress responses, partly because of its antioxidative properties (Kulma and Szopa, 2007). Dopamine also functions in responses to abiotic stresses. When potato (*Solanum tuberosum*) plants are exposed to drought, treatment with abscisic acid or ultraviolet light can significantly increase their concentration of dopamine (Swiedrych et al., 2004). Under salinity stress, activity of tyrosine decarboxylase, a key enzyme in the dopamine synthesis pathway, is enhanced (Swiedrych et al., 2004). In salt-stressed rice (*Oryza sativa*), exogenous dopamine regulates the expression of the aquaporin gene *OsPIP1-3* (Abdelkader et al., 2012). Dopamine can also alleviate salt-induced stress in apple (*Malus hupehensis*) (Li et al., 2015a). However, little is known about its possible role in directing the uptake and resorption of mineral elements by drought-challenged plants.

As one of the most economically important woody plants, commercial apple (*M*. *domestica* Borkh.) is widely cultivated in temperate regions. However, drought has become a source of critical abiotic stress that impacts apple growth, productivity, and geographic distribution. The benefits of dopamine have been documented in studies with salt-induced and nutrient deficiency-induced stress, but no research has focused on the combination of ionome concentrations, uptake, nutrient shifts, partitioning, and resorption under drought stress. Because roots, stems, and leaves show different sensitivities to water deficits, a whole-plant approach is required rather than the traditional emphasis on aboveground organs only. Therefore, our study objective was to determine whether dopamine supplementation could alleviate drought-induced stress in trees of *M*. *domestica* when grown under well-watered and moderate deficit conditions. Our hypothesis was that exogenous dopamine would increase plant tolerance to drought stress. To test this, we assessed (i) photosynthetic responses; (ii) biomass allocations; (iii) ionome concentrations, uptake, shift, partitioning, and resorption in the leaves, stems, and roots; and (iv) the timing of leaf senescence.

## Materials and Methods

### Plant Materials and Growing Conditions

These trials were conducted at the Northwest A&F University, Yangling (34°20′N, 108°24′E), Shaanxi, China, where the climate is semi-arid. In mid-March 2016, buds of cv. ‘Naganofuji No.2’ were grafted onto 1-year-old rootstock of *Malus hupehensis*. All plants were grown in plastic containers (38 × 23 cm) filled with cultivation soil:sand (1:1, v:v). They were located in a greenhouse under ambient light, at 20–35°C, and with a relative humidity of 50–75%. To eliminate position effects, we rotated the containers weekly. Standard horticultural practices were followed for disease and pest control.

### Experimental Design

The experimental layout was completely randomized and consisted of combined watering and dopamine treatments. After 4 months of growth under well-watered conditions, 400 uniform and healthy plants were divided into four groups to render the following regimes (100 plants per treatment): (1) normal control, irrigated daily to maintain 75–85% field capacity (WW); (2) moderate drought, irrigated daily to maintain 45–55% field capacity (DS); (3) dopamine control, irrigated daily to maintain 75–85% field capacity plus 100 μM dopamine (WW + DA); and (4) dopamine combined with moderate drought, irrigated daily to maintain 45–55% field capacity plus 100 μM dopamine (DS + DA). Irrigation was withheld from the drought-stressed plants beginning on 1 July 2016 while normal irrigation continued for the well-watered plants. Transpiration water losses were evaluated gravimetrically by weighing all pots and calculating the changes in weight that occurred between watering events. Afterward, the amount of water lost was added back to each pot every other day at 18:00 h. For half of the plants in either the well-watered or drought treatments, exogenous dopamine was applied with a 100 μM solution replacing the same amount of water added back to the soil every 10 days. To minimize soil evaporation, we covered the soil surface of each pot with a 3-cm-thick layer of sieved sand. The experiments were terminated after 120 days, on 1 November 2016. Plant growth measurements were made on Days 0 and 90, while plant gas exchange, chlorophyll (Chl) concentrations, and gene expression were determined on Days 0, 30, 60, 90, 105, and 120 after the experiments began. Leaf stomata were observed with a JSM-6360LV scanning electron microscope (SEM; JEOL Ltd., Tokyo, Japan) on Day 90, leaf relative water content (RWC) and hydrogen peroxide (H_2_O_2_) were determined on Day 90, and mineral elements were analyzed on Days 0, 90, and 120.

### Growth Measurements

Plant lengths (PLs) were measured from the base of the stem, at soil level, to the terminal bud of the main stem. Trunk diameter (TD) was measured with a digital micrometer (0.001 mm) 10 cm above the graft union. Whole plants from each treatment were harvested and divided into root, stem, and leaf portions. The roots were first rinsed with tap water, and then all samples were washed in tap water, 0.1 mol L^-1^ of HCL, and distilled water. After the total fresh weight (TFW) was recorded, each sample was fixed at 105°C for 15 min, then dried in a forced-air oven at 75°C for 48 h to a constant weight. Total dry weight (TDW) of the biomass was computed as the sum of the values for root, stem, and leaf dry masses. The relative growth rate (RGR) was calculated by the equation of Radford (1967): RGR = (ln DW_2_- ln DW_1_)/(t_2_- t_1_), where DW_1_ is plant dry weight at Day 0 (t_1_), and DW_2_ is plant dry weight at Day 90 (t_2_). Leaf mass fraction (LMF), stem mass fraction (SMF), and root mass fraction (RMF) were calculated as the dry weight of leaf, stem, and root, respectively, divided by the TDW. The root:stem ratio (RSR) was calculated as root dry weight divided by stem dry weight.

### Measurements of Leaf RWC and H_2_O_2_

The leaf RWC was computed according to the method of Gaxiola et al. (2001). Leaves were excised from each treatment and their fresh weights were recorded immediately. After the leaves were floated in deionized water at 4°C overnight, their rehydrated weights were determined. Finally, the leaves were oven-dried at 70°C for 48 h and weighted again. RWC was calculated as follow: RWC = (fresh weight-dry weight)/(rehydrated weight-dry weight). H_2_O_2_ was extracted with 5% (w/v) trichloroacetic acid and measured as described by Patterson et al. (1984).

### Quantification of Gas Exchange and Chlorophyll Concentrations

The net photosynthesis rate (Pn) was monitored with a Li-Cor portable photosynthesis system (Li6400; LICOR, Huntington Beach, CA, United States) on sunny days between 09:00 and 11:00 h. All photosynthetic measurements were taken at 1000 μmol photons m^-2^ s^-1^ and a constant airflow rate of 500 μmol s^-1^. The concentration of cuvette CO_2_ was set at 400 μmol CO_2_ mol^-1^ air. For all treatments, data were recorded from 10 mature, fully exposed leaves from the same position of each selected plant. On each sampling date, Chl was extracted from harvested leaves with 80% acetone, and concentrations were determined spectrophotometrically according to the method of Arnon (1949), using a UV-1750 spectrophotometer (Shimadzu, Kyoto, Japan).

### Observations of Leaf Stomata by SEM

Ten leaves were collected from the same position per treatment. The samples were immediately fixed with a 4% glutaraldehyde solution in 0.1 M phosphate-buffered saline (PBS, pH 6.8) to avoid any damage or alterations during sample preparation. They were first rinsed five times with PBS (for 5, 10, 15, 20, and 30 min), and then dehydrated in a graded ethanol series, vacuum-dried, and gold-coated. Observations were made with the SEM. Stomata were counted at random in 30 visual sections on the abaxial epidermis, and final tallies were used to calculate stomatal density. We used Image J software for measuring stomatal lengths, widths, and apertures.

### Determination of Mineral Elements

After being individually ground and sieved, 0.2-g samples of roots, stems, and leaves were digested with concentrated sulfuric acid (H_2_SO_4_, AR, 98%) and hydrogen peroxide (H_2_O_2_, GR, ≥30%). From the resulting digestion and after the addition of 100 mL of deionized H_2_O, N and P concentrations were obtained with an Auto Analyzer 3 (AA3) continuous flow analyzer (SEAL Analytical, Norderstedt, Germany), while the K concentration was analyzed by a flame photometer (M410; Sherwood Scientific Ltd., Cambridge, United Kingdom). Other 0.1-g samples were digested with nitric acid (HNO_3_, AR, 65%) using the microwave reaction system (Multiwave PRO; Anton Paar GmbH, Graz, Austria). Elemental analyses of S, Ca, Mg, Fe, Mn, Cu, Zn, B, Al, Cr, Ni, As, Mo, Pb, and Cd were performed by inductively coupled plasma-atomic emission spectroscopy (iCAP Q ICP-MS; Thermo Fisher Scientific Co., Waltham, MA, United States).

### Determinations of Nutrient Uptake Fluxes, Shift, Partitioning, and Resorption

Over a 90-day period, nutrient uptake fluxes were calculated based on values for RGR, dry weights, and the total concentrations of nutrients in the root (r), stem (s), and leaf (l), as follows (Kruse et al., 2007; Sanchez-Rodriguez et al., 2010):

(Total Nutrient)_r_ = RGR × DW_r_ × (Nutrient)_r_
(Total Nutrient)_s_ = RGR × DW_s_ × (Nutrient)_s_
(Total Nutrient)_l_ = RGR × DW_l_ × (Nutrient)_l_

Jupt Nutrient = (Total Nutrient)_r_ + (Total Nutrient)_s_ + (Total Nutrient)_l_. The uptake flux was expressed in units of milligrams per plant per day or micrograms per plant per day.

Nutrient transport was defined as the total amount moved to the stems or leaves per root DW per day. Nutrient accumulations in the roots were defined as the total amount of nutrient taken up into the root per root DW per day. The following equation was used for these calculations (Hunt, 1982): transport or accumulation = (M_2_-M_1_) × (LnW_2_- LnW_1_)/(W_2_-W_1_)/(T_2_-T_1_) where M is the total amount of a nutrient in the stem or leaf (transport) or in the root (accumulation), W is the root dry weight, and T is time (here, 90 days). Transport or accumulation was expressed in units of milligrams per gram DW per day or micrograms per gram DW per day.

The total content of a mineral element in a particular organ (root, stem, or leaf) was calculated as the product of DW and concentration in that organ. Partitioning among roots, stems, and leaves was related to the whole-plant content for each nutrient of interest.

Nutrient resorption efficiency (NuRE) was quantified by the following formula (Aerts, 1996): NuRE = ((C_g_ -C_s_)/C_g_) × 100%, where C_g_ and C_s_ are nutrient concentrations in green and senesced leaves, respectively. The nutrient concentrations in senesced leaves were considered direct indicators of nutrient resorption proficiency (NuRP). Therefore, we used NuRP as an index of nutrient conservation in plants because it has been defined as the absolute level to which a nutrient is reduced in senesced leaves (Aerts, 1996).

### RT-PCR Analysis of Expression of *pheide a oxygenase* (*PAO*), and *senescence-associate gene 12* (*SAG12*)

Total RNA was extracted from leaf samples using a Wolact^®^ plant RNA isolation kit (Vicband, Hong Kong, China) according to the manufacturer’s instructions. Quantitative real-time PCR (qRT-PCR) was performed on an ABI StepOnePlus real-time PCR system (Applied Biosystems, Singapore), using SYBR Premix Ex Taq II (Takara, Kyoto, Japan). Gene-specific primers, designed by Primer Premier 6 software (Biosoft International, Palo Alto, CA, United States), were as follows: for *SAG12*, 5′-GAAGGAAGCCATCATTGCAGCCAA-3′ and 5′-ACCATGGTCAAGACTCGTTCCACA-3′; and for *PAO*, 5′-ACCCGAGTGGTTTGGTACTTGTGA-3′ and 5′-TACACGAGGAGCATTTGAGGGTGT-3′. We used *MDH* (5′-CGTGATTGGGTACTTGGAAC-3′ and 5′-TGGCAAGTGACTGGGAATGA-3′) as the endogenous reference to normalize expression. Three independent biological replications were performed for each sample.

### Statistical Analysis

All data were analyzed with SPSS 20.0 software. One-way analysis of variance (ANOVA) was used to compare the means of each treatment. Tukey’s multiple range tests were used at a significance level of *P*_0.05_, and data were presented as the means ± standard deviation (SD) of five to 10 replicate samples. We then applied two-way ANOVA [model: ‘drought,’ ‘dopamine,’ and ‘drought × dopamine], using a general linear model to confirm whether the effects of drought and dopamine, individually and combined, had any significant influence on the results.

## Results

### Growth, Leaf RWC, and H_2_O_2_ Content Determinations

**FIGURE 1:**
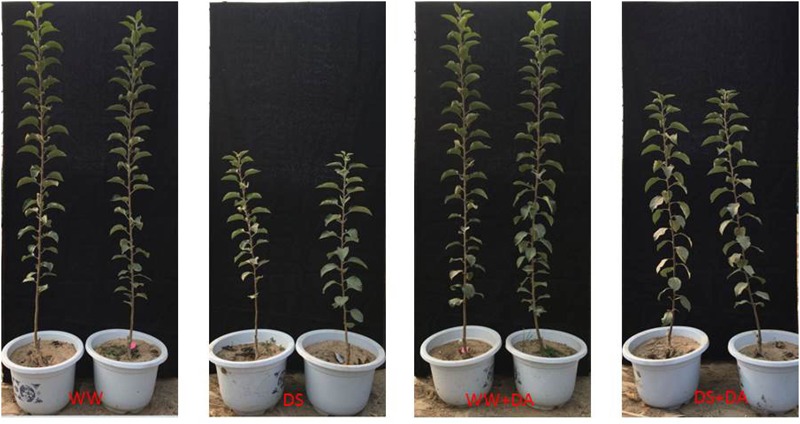
Plants after 90 days of exposure to different watering and dopamine treatments: WW, irrigated daily to maintain 75–85% field capacity; DS, irrigated daily to maintain 45–55% field capacity; WW + DA, irrigated daily to maintain 75–85% field capacity plus 100 μM dopamine; and DS + DA, irrigated daily to maintain 45–55% field capacity plus 100 μM dopamine.

Drought stress had a strong inhibitory effect on overall plant growth, leading to significant decreases in values for PL, TD, TFW, TDW, and RGR of 35.0, 30.3, 55.9, 48.4, and 52.9%, respectively (**Figure 1** and Supplementary Table S1). However, exogenous dopamine significantly alleviated those declines when compared with drought-stressed plants that had received no dopamine, and those respective decreases were then only 25.0, 25.0, 40.0, 29.5, and 28.0% over control values. The interactions between drought and dopamine for these variables were also highly significant. Dry mass allocations were changed in response to stress, with wider variations noted in the root biomass and stem biomass fractions. In general, drought-challenged plants had a higher RMF than the control plants receiving normal irrigation. Although the water deficit decreased the SMF significantly, it had no effect on the LMF. Values for the RSR, which depended to a large extent on the RMF and SMF, were increased significantly due to drought. The influence of dopamine was significant only for the LMF (Supplementary Table S2). After 90 days of stress, the RWC values were significantly lower than the levels measured in well-watered controls. However, the application of dopamine substantially alleviated this response. A significant H_2_O_2_ burst (123.1%) occurred in drought-stressed leaves, but dopamine treatment reduced that burst to 52.1% in similarly stressed plants (Supplementary Table S2).

### Net Photosynthesis and Chlorophyll Concentrations

**FIGURE 2:**
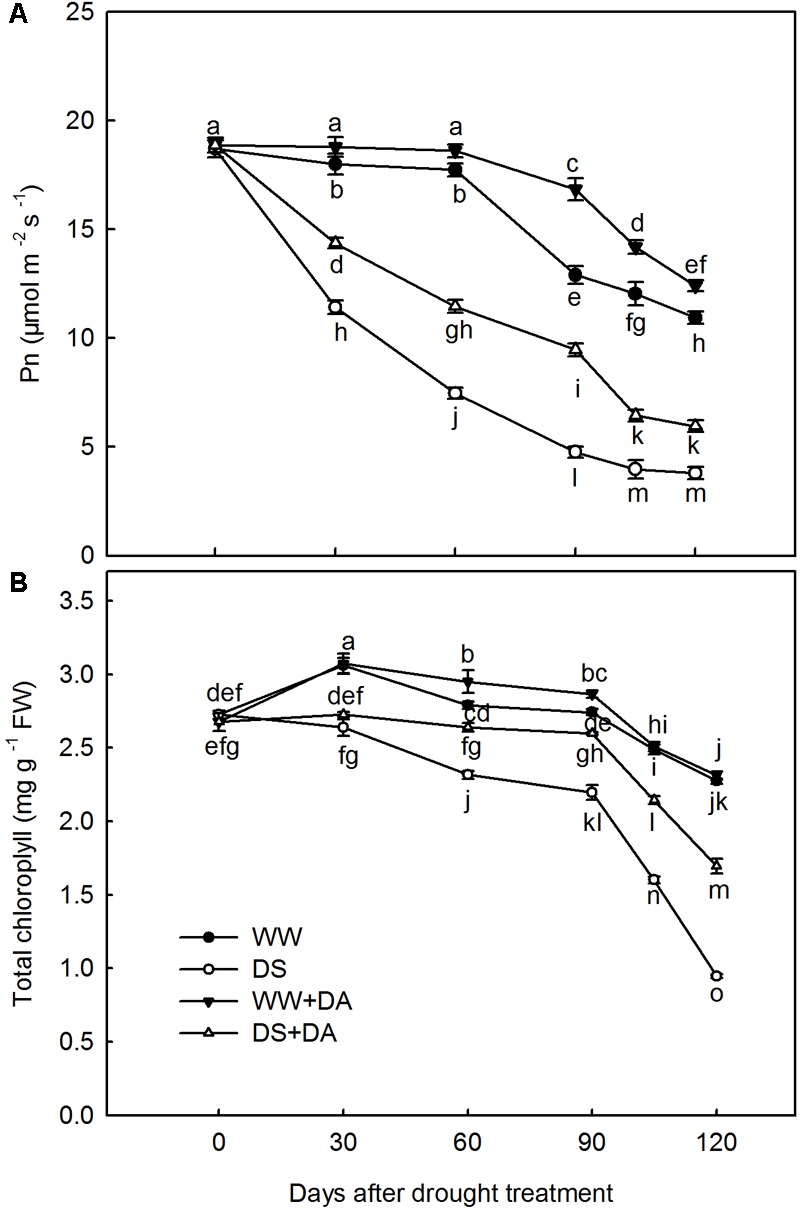
Effects of dopamine on **(A)** net photosynthesis rate (Pn) and **(B)** total chlorophyll concentrations. Data are means ± SD of 10 replicate samples. An ANOVA test followed by Tukey’s multiple range test was performed. Time points not labeled with the same letter indicate significant differences at *P_0.05_* level. Treatments: WW, irrigated daily to maintain 75–85% field capacity; DS, irrigated daily to maintain 45–55% field capacity; WW + DA, irrigated daily to maintain 75–85% field capacity plus 100 μM dopamine; and DS + DA, irrigated daily to maintain 45–55% field capacity plus 100 μM dopamine.

In response to drought stress, Pn decreased in all treatments throughout the experimental period, with rates being significantly lower for no-dopamine than for dopamine-applied plants. On Day 120, Pn from dopamine-applied plants were 1.15 and 1.57 times higher than the rate for well-watered and drought-stressed control plants, respectively (**Figure 2A**). After 120 days of stress, total Chl concentrations were significantly lower than the levels measured in well-watered controls. In particular, total Chl was reduced by 58.4 and 25.4% in no-dopamine and dopamine-applied plants, respectively (**Figure 2B**).

### Stomatal Behavior

**FIGURE 3:**
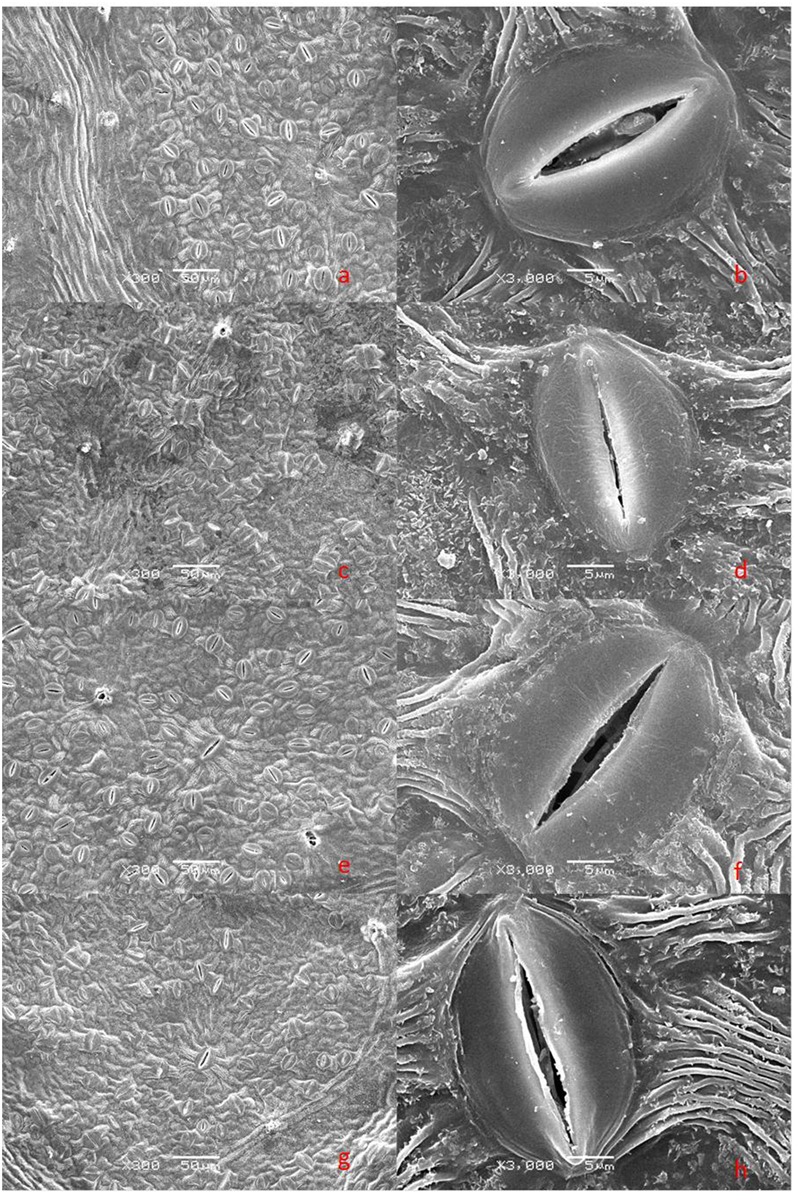
Scanning electron microscope (SEM) images of stomata from plants after 90 days of exposure to different watering and dopamine treatments. **(a,b)** WW, irrigated daily to maintain 75–85% field capacity; **(c,d)** DS, irrigated daily to maintain 45–55% field capacity; **(e,f)** WW + DA, irrigated daily to maintain 75–85% field capacity plus 100 μM dopamine; and **(g,h)** DS + DA, irrigated daily to maintain 45–55% field capacity plus 100 μM dopamine. **(a,c,e,g)** magnification × 300, scale bars = 50 μm; **(b,d,f,h)** magnification × 3000, scale bars = 5 μm.

**FIGURE 4:**
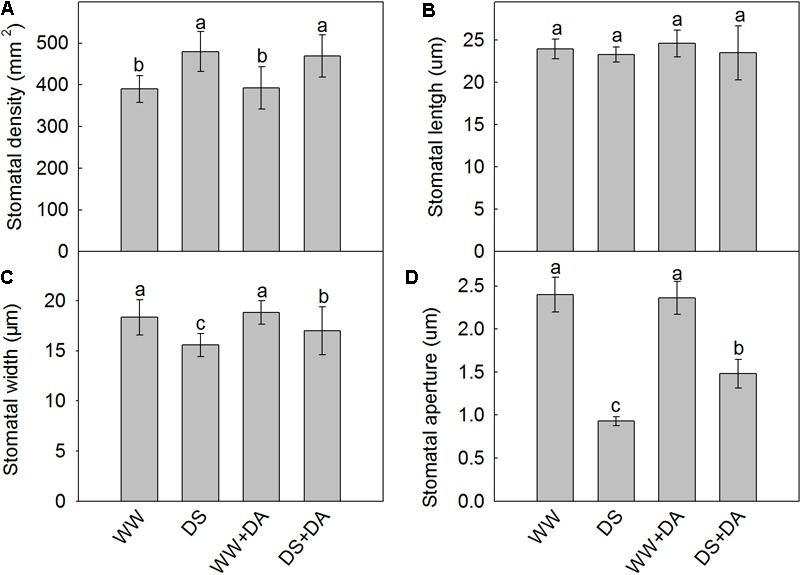
Properties of stomata from plants after 90 days of exposure to different watering and dopamine treatments. **(A)** Density, **(B)** length, **(C)** width, and **(D)** aperture size. Data are means ± SD of 30 images. An ANOVA test followed by Tukey’s multiple range test was performed. For each panel, bars not labeled with same letter indicate significant differences at *P_0.05_* level. Treatments: WW, irrigated daily to maintain 75–85% field capacity; DS, irrigated daily to maintain 45–55% field capacity; WW + DA, irrigated daily to maintain 75–85% field capacity plus 100 μM dopamine; and DS + DA, irrigated daily to maintain 45–55% field capacity plus 100 μM dopamine.

The lower surfaces of the leaf samples were scanned at 300× and 3000× magnifications (**Figure 3**). Drought stress changed values for the stomatal parameters, and clear structural differences were observed between treatments with or without dopamine. Stomatal density was higher in drought-stressed leaves than in the control, but the stomatal widths and stomatal apertures of the former type were significantly decreased. Although exogenous dopamine had no marked effect on stomatal density and stomatal lengths, it did increase stomatal widths and stomatal apertures significantly under drought conditions (**Figure 4**).

### Mineral Nutrient Concentrations in Plant Tissues

The concentrations of mineral nutrients measured in leaves after 90 days of treatment are shown in Supplementary Table S3. Drought conditions were associated with significant reductions in the levels of N, P, K, S, Cu, B, As, and Mo but increases in Ca, Mg, Mn, Ni, and Pb concentrations. Stress had no critical influence on Fe, Zn, Al, Cr, or Cd concentrations. Under drought conditions, exogenous dopamine significantly increased concentrations of N, P, K, Cu, and Pb by a range of 4.1% (for N) to 20.0% (Pb) but led to marked declines for Ca, Mg, Mn, and Al by 12.8% (Mn) to 24.5% (Al) in dopamine-applied plants when compared with no-dopamine applied plants.

Stress also altered the concentrations of mineral nutrients in the stems (Supplementary Table S4), diminishing the levels of P, Mn, Cu, B, Cr, Ni, As, and Mo, enhancing the amounts of N, K, Ca, S, Zn, and Cd, but having no significant influence on Mg, Fe, or Al. Under drought conditions, exogenous dopamine increased the concentrations of P, Cu, Zn, and Al by 8.9% (Cu) to 38.2% (Zn); reduced the levels of Ca, Mg, S, Fe, Pb, and Cd by 14.9% (Mg) to 64.3% (Pb), but had no significant effect on N, K, Mn, B, Cr, Ni, As, or Mo.

In roots during the drought period, the concentrations of P, K, Cu, Zn, B, and As were reduced while those of N, Fe, Al, Cr, Mo, Pb, and Cd were increased (Supplementary Table S5). No significant differences in the levels of Ca, Mg, S, Mn, or Ni were found between stressed plants and well-watered plants. Under drought conditions, the comparison between no-dopamine and dopamine-applied plants showed that the addition of this molecule increased the concentration of P by 18.2% but reduced the levels of N, Ca, Mg, S, Fe, Mn, Cu, Zn, Al, As, Mo, Pb, and Cd by 3.6% (N) to 55.4% (Cd), while having no significant effect on K, B, Cr, or Ni.

### Uptake Fluxes in Mineral Nutrients

Data for mineral nutrient uptake are presented in Supplementary Table S6. When comparing between well-watered and drought-stressed plants, reductions of 46.8% (for Cd) to 81.7% (P) were noted for all of the elements analyzed here. However, exogenous dopamine was associated with significant increases in the uptake of these nutrients under both well-watered and drought conditions. When the comparison was made between dopamine and no-dopamine plants under stress, uptake of all of those nutrients was improved by 2.4% (Cd) to 151.8% (P).

### Transport of Mineral Nutrients to Leaves and Stems and Their Accumulations in Roots

Drought stress sharply reduced the rate of transport for all analyzed nutrients, with reductions ranging from 15.1% (for Ca) to 292.4% (Cr) (Supplementary Table S7). However, the addition of dopamine altered those rates under drought conditions, leading to increases in the transport of N, P, K, S, Cu, Zn, B, Ni, Mo, and Pb (range of 4.6% for S to 527.4% for Mo) but decreases in the transport of Ca, Mg, Fe, Mn, Al, Cr, As, and Cd (range of 4.9% for Fe to 35.6% for Al).

Transport of nutrients to the stem was also decreased by drought stress for all analyzed elements, with rate reductions ranging from 33.0% (S) to 281.6% (Cr) (Supplementary Table S8). When comparing dopamine versus no-dopamine plants under drought conditions, the transport rate was improved for 15 elements, with increases ranging from 6.0% (Cd) to 228.1% (Al). The three exceptions were Ca, S, and Pb, for which their transport rates into the stem were reduced by 4.9, 17.5, and 122.6%, respectively.

When compared with the well-watered treatment, drought stress caused higher root accumulations of N, S, Fe, Mn, Al, Mo, and Pb (increases ranging from 11.8% for Al to 301.2% for Pb) but marked declines in the accumulation of P, K, Ca, Mg, Cu, Zn, B, Cr, Ni, As, and Cd (reductions ranging from 2.6% for Cd to 107.1% for Ni) (Supplementary Table S9). Under drought conditions, exogenous dopamine significantly increased root accumulations of N, P, K, Fe, Zn, B, and Cr (range of 11.1% for B to 176.3% for P), but reduced those rates for Ca, Mg, S, Mn, Cu, Al, Ni, As, Mo, Pb, and Cd (7.1% for Ni to 151.5% for Pb).

### Partitioning of Mineral Nutrients Within the Whole Plant

**FIGURE 5:**
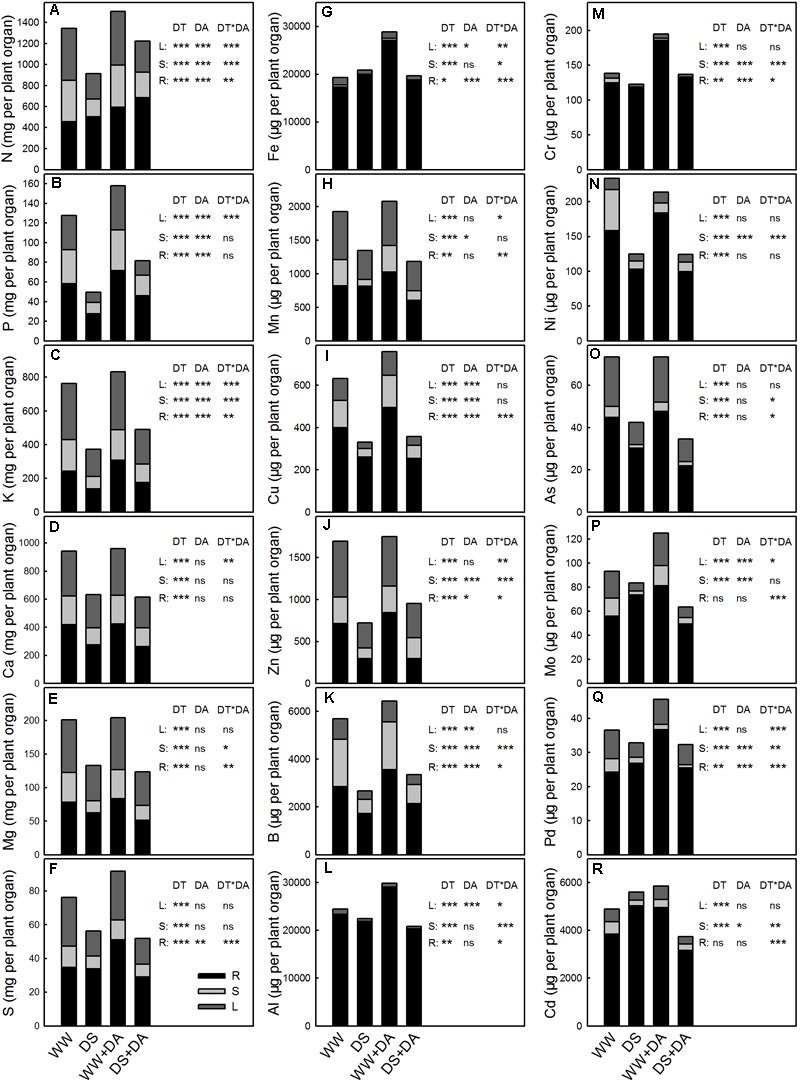
Content and partitioning of elements in and between roots (R), stems (S), and leaves (L) of plants after 90 days of exposure to different water and dopamine conditions. **(A)** Nitrogen, **(B)** Phosphorus, **(C)** Potassium, **(D)** Calcium, **(E)** Magnesium, **(F)** Sulfur, **(G)** Iron, **(H)** Manganese, **(I)** Copper, **(J)** Zinc, **(K)** Boron, **(L)** Aluminum, **(M)** Chromium, **(N)** Nickel, **(O)** Arsenic, **(P)** Molybdenum, **(Q)** Plumbum, and **(R)** Cadmium. Treatments: WW, irrigated daily to maintain 75–85% field capacity; DS, irrigated daily to maintain 45–55% field capacity; WW + DA, irrigated daily to maintain 75–85% field capacity plus 100 μM dopamine; and DS + DA, irrigated daily to maintain 45–55% field capacity plus 100 μM dopamine. Significant effects of the main factors drought (DT), dopamine (DA) and the interactions (DT × DA) are also given in the figure: ns, not significant; ^∗^*P* < 0.05; ^∗∗^*P* < 0.01; and ^∗∗∗^*P* < 0.001.

Nutrient contents were generally the highest in the roots, although they varied widely. Significant differences across treatments in the partitioning of elements were observed, and drought conditions increased that partitioning (**Figure 5**). When comparing well-watered versus drought stress, the largest change in partitioning within the roots was noted for Mo (59.9–88.1%) while the smallest difference was found for K (31.8–36.8%). In the leaves, partitioning was greatly reduced in response to the water deficit, ranging from 23.8% down to 8.04% for Mo to 14.9 to 13.5% for B. Exogenous dopamine also affected whole-plant partitioning, enhancing the leaf and stem accumulations of most analyzed elements. When considering no-dopamine versus dopamine treatment, changes in nutrient levels of root ranged from 88.1 to 77.8% for Mo down to 97.3 to 97.2% for Al.

### Resorption Proficiency and Efficiency of Mineral Nutrients

We found that Ca, Fe, Cu, Zn, Al, As, Pb, and Cd were more highly concentrated in senesced leaves than in green leaves while the opposite was noted for N, P, K, Mg, S, Mn, B, Cr, Ni, and Mo (Supplementary Tables S10, S11). The NuRP values for all analyzed elements except N, K, Mg, and Mn were reduced by drought stress. However, exogenous dopamine improved the resorption proficiency of all elements in drought-stressed plants.

**FIGURE 6:**
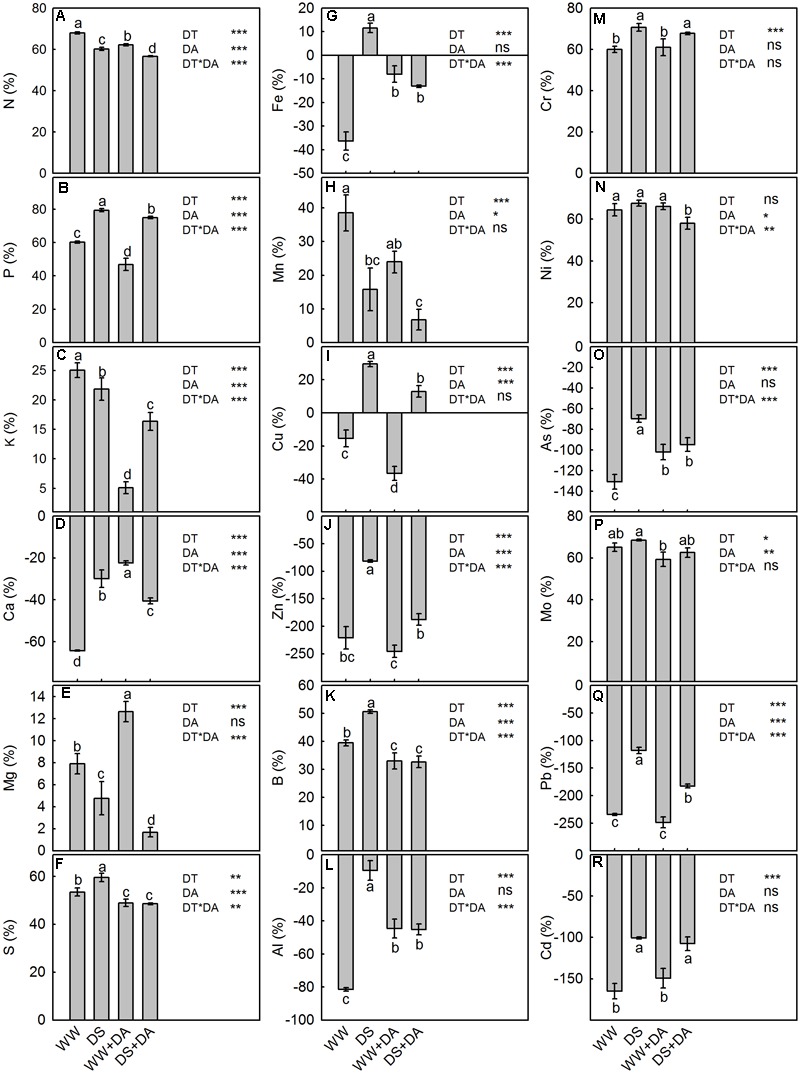
Nutrient resorption efficiency of elements in plants after 120 days of exposure to different watering and dopamine conditions. **(A)** Nitrogen, **(B)** Phosphorus, **(C)** Potassium, **(D)** Calcium, **(E)** Magnesium, **(F)** Sulfur, **(G)** Iron, **(H)** Manganese, **(I)** Copper, **(J)** Zinc, **(K)** Boron, **(L)** Aluminum, **(M)** Chromium, **(N)** Nickel, **(O)** Arsenic, **(P)** Molybdenum, **(Q)** Plumbum, and **(R)** Cadmium. Data are means ± SD of 5 replicate samples. An ANOVA test followed by Tukey’s multiple range test was performed. Different letters in the column represent significant difference at *P_0.05_* level. Significant effects of the main factors drought (DT), dopamine (DA) and the interactions (DT × DA) are also given in the figure: ns, not significant; ^∗^*P* < 0.05; ^∗∗^*P* < 0.01; and ^∗∗∗^*P* < 0.001. Treatments: WW, irrigated daily to maintain 75–85% field capacity; DS, irrigated daily to maintain 45–55% field capacity; WW + DA, irrigated daily to maintain 75–85% field capacity plus 100 μM dopamine; and DS + DA, irrigated daily to maintain 45–55% field capacity plus 100 μM dopamine.

The efficiency by which plants resorbed mineral nutrients from senesced leaves was positive for all elements except Ca, Fe, Cu, Zn, Al, As, Pb, and Cd (respective negative NuRE values of 64.3, 36.4, 15.4, 220.8, 81.5, 130.6, 234.5, and 165.0%). This compared to mean positive NuRE values for N, P, K, Mg, S, Mn, B, Cr, Ni, and Mo of 68.0, 60.3, 25.0, 7.9, 53.4, 38.5, 39.5, 60.0, 64.4, and 65.2%, respectively (**Figure 6**). Except for Ni, drought stress had a significant influence on NuRE, with increases ranging from 3.3% (Mo) to 98.4% (Zn). Exogenous dopamine had a positive effect on the amount of Ca resorbed NuRE mean value, but it was associated with decreased mean NuRE values for N (4.7%), P (8.9%), K (12.7%), S (7.8%), Mn (11.8%), Cu (18.9%), Zn (65.3%), B (12.2%), Ni (4.0%), Mo (5.9%), and Pb (39.5%). This molecule had no observable influence on the NuRE of Mg, Fe, Al, Cr, As, or Cd.

### Effects of Drought Stress and Dopamine on the Relative Expression of Genes for Leaf Senescence

**FIGURE 7:**
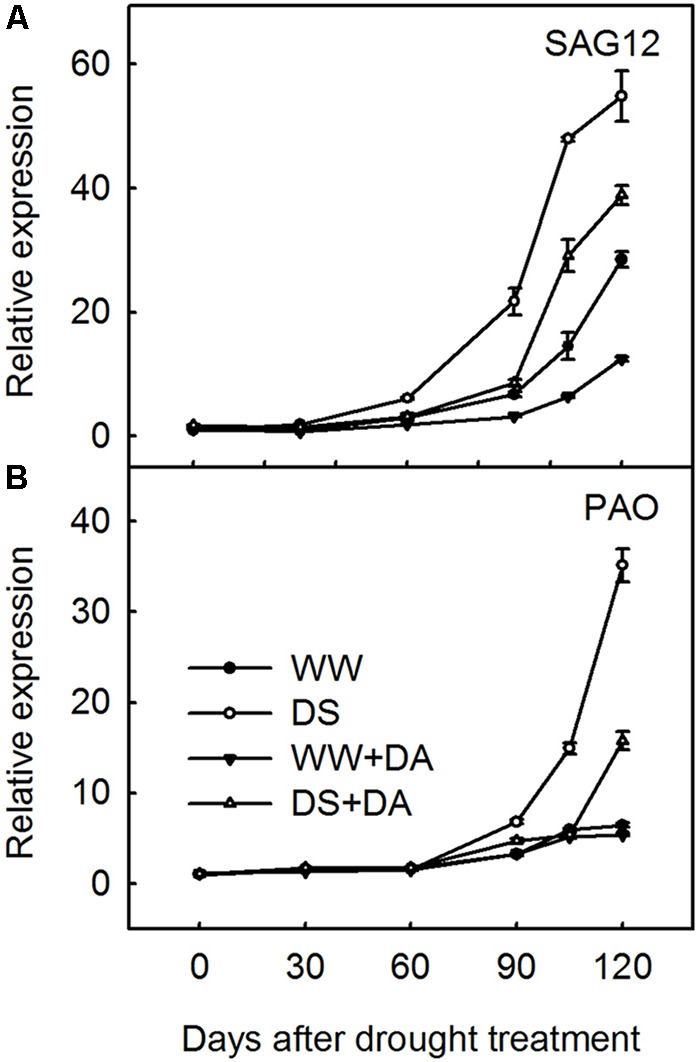
Effects of dopamine on expression of SAG12 **(A)** and PAO **(B)** in leaves under different treatment conditions. Total RNA was isolated from samples at specified time points (0–120 days), converted to cDNA, and subjected to qRT-PCR. Expression levels were calculated relative to expression of *Malus MDH* mRNA. Data are means ± SD of 5 replicate samples. Treatments: WW, irrigated daily to maintain 75–85% field capacity; DS, irrigated daily to maintain 45–55% field capacity; WW + DA, irrigated daily to maintain 75–85% field capacity plus 100 μM dopamine; and DS + DA, irrigated daily to maintain 45–55% field capacity plus 100 μM dopamine.

Over our 120-day experimental period, relative expression of *SAG12* and *PAO* was significantly up-regulated in leaves from drought-stressed plants when compared with those from well-watered plants (**Figure 7**). However, when dopamine was applied to either well-watered or droughty soils, the relative expression of these genes was obviously inhibited, especially in leaves from stressed plants. By Day 120, the relative expression abundance of *SAG12* and *PAO* in DS + DA leaves was decreased by 29.2 and 55.1%, respectively, when compared with the corresponding DS plants. This demonstrated therefore that long-term exogenous application of 100 μM dopamine to the soil could retard drought-induced leaf senescence.

## Discussion

### The Effect of Drought Stress and Dopamine on Plant Growth and Biomass Allocations

Plant growth is affected by drought in several ways, e.g., reduced leaf water potential, altered plant water and nutrient relationships (Boomsma and Vyn, 2008). Our data also indicated that, although drought treatments led to a decline in many parameters, exogenous applications of dopamine eased those inhibitory effects. The interaction between drought and dopamine indicated that the responses of these growth parameters to watering regime (well-watered or drought stressed) were significantly influenced by the application of dopamine. The trend was similar for biomass allocation, RWC and H_2_O_2_, with drought, dopamine, and their interaction being significant for all parameters measured, except drought was not significant for LMF, dopamine and their interaction were not significant for RMF, SMF, and RSR. This molecule can function as an antioxidant and is efficient in scavenging free radicals due to its reduction power (Kanazawa and Sakakibara, 2000). Therefore it can enhance growth and development under different scenarios (Endress et al., 1984). For example, we have reported that, under salt-induced stress conditions, pretreatment with dopamine significantly suppresses the production of H_2_O_2_ and increases plant height, fresh and dry weights, and RSR in *Malus* seedlings (Li et al., 2015a). We have also demonstrated that, under a nutrient deficiency, growth of *M*. *hupehensis* plants is noticeably inhibited, but treatment with exogenous dopamine markedly alleviates that inhibition (Liang et al., 2017). Dopamine is coordinated with phytohormone activity to regulate growth and enable plants to fine-tune their stress responses (Kulma and Szopa, 2007). Dopamine has also been identified as a key factor in the growth of *Lactuca sativa* hypocotyls, and its level in potato plants is also significantly increased under drought conditions (Swiedrych et al., 2004). We found that exogenous dopamine significantly increased growth parameters and helped reverse the negative influence of drought stress in *Malus*.

### The Effect of Drought Stress and Dopamine on Net Photosynthesis, Chlorophyll Concentrations, and Stomatal Behavior

We noted a concomitant decrease in both the rate of photosynthesis and behavior of the stomatal apertures, as well as lower Chl concentrations, all of which suggest that the stomata and Chl have critical roles in inhibiting photosynthetic activity under drought conditions (Li et al., 2015b). However, in the presence of dopamine, the rate of photosynthesis was maintained at a higher level throughout the experimental period when compared with plants that did not receive that exogenous application. After 120 days of drought treatment, the stomatal apertures and Chl levels were higher in leaves from dopamine-treated plants. This might explain why plants in that treatment group were better able to maintain higher photosynthetic values under stress. Therefore, even though photosynthesis was reduced under drought conditions, exogenous supplementation with dopamine significantly eased those inhibitory effects by maintaining stable stomatal apertures and photosynthetic pigments. In addition to the supporting results that we have already published (Li et al., 2015a; Liang et al., 2017), Kulma and Szopa (2007) have shown that dopamine can control the reduction in photosynthesis in isolated *Spinacia oleracea* chloroplasts by functioning as a chemical analog of a proposed natural mediator, or oxygen-reducing factor, that allows for energy transduction during photosynthesis. Dopamine also can directly or indirectly mitigate salt-induced and nutrient deficiency-induced restrictions to photosynthetic performance (Li et al., 2015a; Liang et al., 2017).

### The Effect of Drought Stress on Nutrient Concentrations and Their Uptake, Transport, Partitioning, and Resorption

Because drought stress reduces both the total accumulation of dry matter and nutrient uptake, the final concentrations of nutrients in tissues from plants grown under a water deficit will depend upon the relative reduction in nutrient absorption relative to the reduction in dry matter accumulation (Samarah et al., 2004). The water supply is a critical variable that controls nutrient absorption, and drought can inhibit plant growth by reducing the uptake, transport, and redistribution of elements (Sardans and Penuelas, 2012). Such stress inhibits nutrient absorption not only by reducing nutrient supply through mineralization but also by decreasing nutrient diffusion and mass flow in the soil (Sanaullah et al., 2012). Similar to the results reported by other researchers (Omirou et al., 2013; Zhao et al., 2015), our data revealed that drought stress significantly diminished the uptake of nutritional elements by these apple plants. This might explain their lower growth rate which led to reduced biomass accumulations due to the negative impact of drought on assimilation capacity (Ludlow and Muchow, 1990). He and Dijkstra (2014) have indicated that drought stress negatively affects plant nutrition because the reduction in nutrient uptake is larger than the reduction in plant growth. Similarly, nutrient uptake plays a vital role in conferring plant tolerance to a water deficit (Samarah et al., 2004). Moisture stress inhibits the flow of nutrients within the soil, the uptake of those elements, and their absorption by the plants (Fageria et al., 2002). Drought conditions prevent the absorption of macro- and micronutrients by cherry tomato plants (Sanchez-Rodriguez et al., 2010). This stress also prevents the uptake of N, P, Fe, Mn, Zn, and other ions in plants, causing symptoms of mineral deficiency (Sardans and Penuelas, 2012; Yasar et al., 2014).

In addition to studying nutrient concentrations and uptake in relation to drought scenarios, one must consider the long-distance transport and partitioning of nutrients if we are to improve our understanding of how plants function under drought conditions. Less transpiration due to drought leads to a reduction in volume flow in the xylem, which then restricts transport and decreases mass flow and diffusivity of nutrients between the roots and stems (Kramer and Boyer, 1995; He and Dijkstra, 2014). Therefore, drought stress ultimately change the overall distribution of nutrients that can be absorbed by plant roots in the soil (Alam, 1999). Because leaves, stems, and roots generally differ in their sensitivity to drought, it is better to take a whole-plant approach rather than focusing only on certain organs when examining the influences of this stress on tree functions (Peuke and Rennenberg, 2011). Although drought conditions greatly influence the rate at which N, P, and K (and other nutrients) are transported to the leaves and stems, the rate at which the first three are accumulated in the roots is sharply increased under a water deficit (Wang et al., 2009). In beech seedlings, drought stress affects the partitioning of Ca, Mn, and Al among the roots, stems, and leaves (Peuke and Rennenberg, 2011). We found here that the rate of nutrient transport to the leaves and stems was reduced in stressed plants, and that the rate of accumulation for most of the analyzed elements was decreased in the roots of those stricken plants. Moreover, the percent reduction was higher in the leaves than in the stems or roots. This might have been due to the leaf being very sensitive to drought. Although nutrient contents were lower in all three tissue types, the roots contained the highest levels. Furthermore, drought treatment enhanced the partitioning of elements in the roots, as reflected by higher values calculated for the RMF and RSR. Plants can regulate their growth, morphology, and physiochemical characteristics when adapting to drought stress, enabling the roots to obtain more water and nutrients from deeper in the soil (Turner and Haygarth, 2001). Thus, nutrient uptake under drought stress might be further improved by increasing the RSR under such conditions (Chapin et al., 1993).

Resorption is an important physiological means for retaining nutrients in plants, and is a dynamic, highly regulated process that involves the exchange of nutrients and metabolites between organs (Freschet et al., 2010). This conservation mechanism can also affect key components of plant growth, such as nutrient uptake and interspecific competition (Aerts, 1996). Although nutrient resorption potentially can occur year-round, it is most pronounced during periods of organ senescence, when it includes all senescing plant parts and leads to total dormancy (Gordon and Jackson, 2000). This phenomenon can be quantified by two parameters (Aerts, 1996): NuRE, defined as the proportion of a nutrient that is resorbed from the leaf before its abscission; and NuRP, which is the final nutrient concentration in senesced leaves (van Heerwaarden et al., 2003). Nutrients that are resorbed and stored internally, rather than lost through leaf fall, are a readily available pool for plants (Marchin et al., 2010). Nutrient conservation and the chemical concentration of plant litter are regulated both by the initial production and the subsequent resorption of metabolites, with environment affecting both of those processes differentially (Suseela et al., 2015). Improving our knowledge about nutrient resorption would provide a great deal of information regarding strategies for nutrient use.

The surrounding environment regulates the chemical makeup of plant litter not only through its direct effect on the biosynthesis of metabolites during the growing season, but also through the metabolites that are obtained during tissue senescence (Tharayil et al., 2011; Suseela et al., 2015). In fact, nutrient resorption is sometimes influenced more strongly by environment than by genetics (Agren and Weih, 2012). One key factor is water availability (Killingbeck, 1993). Drought stress affects plant phenology, sink–source relationships, and patterns of leaf abscission and phloem transport, all of which can possibly alter nutrient resorption (Estiarte and Penuelas, 2015). Although a water deficit can lead to the early onset of senescence that potentially increases resorption, severe drought stress causes damage to phloem transport and the loading of photosynthates, which significantly decreases nutrient resorption (Pugnaire and Chapin, 1992). We found here that, except for N, K, Mg, and Mn, the NuRP values were reduced for all analyzed elements in stressed plants. Whereas NuRE values were increased by drought for P, K, Ca, S, Fe, Cu, Zn, B, Al, Cr, As, Mo, Pb, and Cd, they were decreased for N, Mg, and Mn. For plants that are adapted to drier habitats, drought conditions are associated with low NuRE and higher NuRP values for nitrogen (Khasanova et al., 2013). However, drought stress can also diminish the NuRE of nitrogen in some plants, suggesting that drought-induced leaf senescence leads to a net loss of nutrients (Killingbeck et al., 1990). When NuRE is enhanced by drought conditions (Suseela et al., 2015), those higher NuRE values and low NuRP enable plants to re-use their internally stored nutrients rather than losing them to the leaf litter, thus promoting plant growth, reproduction, fitness, and competitiveness (Aerts and Chapin, 2000).

### The Effect of Dopamine on Nutrient Concentrations, Uptake, Transport, Partitioning, and Resorption

We have previously reported that the capacity of plants to tolerate salt stress is due to the positive impact that exogenous dopamine has on ion uptake (Li et al., 2015a). Such applications significantly increase the concentrations of N, P, K, S, Cu, and Mn under saline conditions. In this research, the dopamine and drought × dopamine interaction were significant for minerals N, P, K, Ca, Mg, and Mo in leaves, Ca, Mg, S, Zn, Cr, and Ni in stems, and N, Ca, Mg, Mn, Cu, As, Mo, and Cd in roots. Furthermore, the concentrations, uptake, and transport of macro- and microelements are decreased by nutrient deficiency-induced stress, but those inhibitions are not as severe when exogenous dopamine is part of the treatment (Liang et al., 2017). Likewise, we showed here that supplemental dopamine promoted the concentration, uptake, and transport of nutrients under drought conditions, but had a negative effect on nutrient resorption. And the interaction between the drought and dopamine treatment was significant for the uptake of N, K, Ca, Mg, S, Fe, Cu, Zn, Mo, and Cd. A significant crossover interaction is also evident for nutrient transport, partitioning and resorption among the two treatments. Such as the interaction between drought and dopamine was significant for the NuRE of N, P, K, Ca, Mg, S, Fe, Zn, B, Al, Ni, As, and Pb.

The positive influence on nutrient uptake and the negative effect on resorption might have resulted because the dopamine application delayed leaf senescence under the water deficit. These responses were manifested in several ways, e.g., reduced occurrence of leaf-yellowing, which is connected with the degradation of chlorophyll and chlorophyll–protein complexes. Although such degradation will progress throughout the senescence phase, it is accelerated by drought stress (Wang et al., 2013). As the key chlorophyll degradation gene, *PAO* encodes for a Fe-dependent monooxygenase located in the envelope membrane of gerontoplasts (Hortensteiner, 2006). Microarray analysis using the Genevestigator tool has shown that *PAO* is up-regulated in response to various environmental stresses, such as pathogen infection or osmotic stress, and that response coincides with Chl breakdown under such conditions (Thomas et al., 2001). Our data for total Chl concentrations and *PAO* expression clearly indicated that exogenous dopamine slowed the degradation in leaves from stressed plants. We also employed another senescence-related gene, *SAG12*, to monitor the senescence process and discovered that its expression pattern closely resembled that of *PAO.* Therefore, we believe that dopamine can delay senescence, thereby influencing nutrient uptake and resorption.

## Conclusion

We studied the effects of applying exogenous dopamine to water-depleted soil and investigated its long-term effects on nutrient status and leaf senescence under drought conditions. When stressed apple plants received supplemental dopamine (100 μM), they exhibited improved growth and photosynthesis. This molecule helped regulate chlorophyll concentrations and stomatal behavior, while also altering the uptake, transport, partitioning, and resorption of nutrients within the whole plant. Our qRT-PCR results showed that the addition of dopamine significantly delayed the process of drought stress-induced leaf senescence. We propose that this anti-senescence, regulatory role of dopamine has a positive influence on drought tolerance and offers new opportunities for its use in agriculture, especially in regions that are challenged by such stress conditions in the field.

## Author Contributions

FM, ChL, and BL conceived and designed the experiments. BL performed the experiments with assistance from TG, QZ, CM, QC, ZW, and CuL. BL performed the data analyses and wrote the manuscript. FM and ChL provided financial support and helped perform the analysis with constructive discussions.

## Conflict of Interest Statement

The authors declare that the research was conducted in the absence of any commercial or financial relationships that could be construed as a potential conflict of interest.
