# Supplementary material for: Effects of Exogenous Dopamine on the Uptake, Transport, and Resorption of Apple Ionome Under Moderate Drought
Source: Front Plant Sci. 2018 Jun 5;9:755. doi: 10.3389/fpls.2018.00755 (PMC5996283; doi:10.3389/fpls.2018.00755)
Supplement: Supplementary file 1 [file Table_1.pdf]

Effects of exogenous dopamine on the uptake, transport, and resorption of apple ionome under moderate drought

Bowen Liang, Tengteng Gao, Qi Zhao, Changqing Ma, Qi Chen, Zhiwei Wei, Cuiying Li, Chao Li*, Fengwang Ma*

State Key Laboratory of Crop Stress Biology for Arid Areas/Shaanxi Key Laboratory of Apple, College of Horticulture, Northwest A&F University, Yangling 712100, Shaanxi, China

***Corresponding authors:** Chao Li, Fengwang Ma
E-mail addresses: lc453@163.com (C. Li), fwm64@nwsuaf.edu.cn & fwm64@sina.com (F. Ma)

TABLE S1 | Plant length (PL), trunk diameter (TD), total fresh weight (TFW), total dry weight (TDW), and relative growth rate (RGR) of plants after 90 d under different watering and dopamine treatments. Data are means ± SD (n=10). An ANOVA test followed by Tukey's multiple range test was performed. Within a column, values not followed by the same letter indicate significant differences at $P_{0.05}$ level. Significant effects of the main factors drought (DT), dopamine (DA) and the interactions (DT×DA) are also given in the table: ns, not significant; *, P <0.05; **, P <0.01; and ***, P <0.001. Treatments: WW, irrigated daily to maintain 75-85% field capacity; DS, irrigated daily to maintain 45-55% field capacity; WW+DA, irrigated daily to maintain 75-85% field capacity plus 100 μM dopamine; and DS+DA, irrigated daily to maintain 45-55% field capacity plus 100 μM dopamine.

Treatment	PL (cm)	TD (mm)	TFW (g $plant^{-1}$)	TDW (g $plant^{-1}$)	RGR (g kg^{-1} d^{-1})
WW	143.28±4.57a	10.45±0.31a	264.69±7.18a	105.30±4.91a	13.91±0.52a
DS	93.16±5.27c	7.28±0.28c	116.70±5.62c	54.35±3.73c	6.55±0.76c
WW+DA	144.88±4.20a	10.54±0.28a	270.49±4.93a	109.91±5.24a	14.38±0.54a
DS+DA	107.40±5.41b	7.84±0.41b	158.96±5.16b	74.20±2.95b	10.02±0.43b
			Significance of effects		
DT	***	***	***	***	***
DA	***	***	***	***	***
DT×DA	***	***	***	***	***

TABLE S2 | Root mass fraction (RMF), stem mass fraction (SMF), leaf mass fraction (LMF), root stem ratio (RSR), relative water content (RWC), and H_2O_2 content of plants grown for 90 d under different watering and dopamine treatments. Data are means ± SD (n=10). An ANOVA test followed by Tukey's multiple range test was performed. Within a column, values not followed by the same letter indicate significant differences at $P_{0.05}$ level. Significant effects of the main factors drought (DT), dopamine (DA) and the interactions (DT×DA) are also given in the table: ns, not significant; *, P <0.05; **, P <0.01; and ***, P <0.001. Treatments: WW, irrigated daily to maintain 75-85% field capacity; DS, irrigated daily to maintain 45-55% field capacity; WW+DA, irrigated daily to maintain 75-85% field capacity plus 100 μM dopamine; and DS+DA, irrigated daily to maintain 45-55% field capacity plus 100 μM dopamine.

Treatment	RMF (%)	SMF (%)	LMF (%)	RSR	RWC (%)	H_2O_2 (μmol g^{-1} FW)
WW	50.39±2.35b	30.99±1.97a	18.62±1.19a	1.64±0.17b	91.33±0.18a	1.17±0.11c
DS	57.83±3.18a	22.61±1.98b	19.56±1.73a	2.59±0.36a	86.62±0.21c	2.61±0.09a
WW+DA	51.51±2.15b	30.30±1.59a	18.20±1.06ab	1.71±0.15b	91.45±0.12a	1.07±0.07c
DS+DA	59.73±2.07a	23.48±1.54b	16.79±0.97b	2.56±0.26a	88.91±0.41b	1.78±0.08b
			Significance of effects			
DT	***	***	ns	***	***	***
DA	ns	ns	***	ns	***	***
DT×DA	ns	ns	**	ns	***	***

TABLE S3 | Concentration of nutritional elements in leaf dry matter from plants grown for 90 d under different watering and dopamine treatments. Data are means ± SD (n=5). Unit of measure: mg g^{-1} DW for N, P, K, Ca, Mg, and S; µg g^{-1} DW for Fe, Mn, Cu, Zn, B, Al, Cr, Ni, As, Mo, Pb, and Cd. An ANOVA test followed by Tukey's multiple range test was performed. Within a row, values not followed by the same letter indicate significant differences at $P_{0.05}$. level. Significant effects of the main factors drought (DT), dopamine (DA) and the interactions (DT×DA) are also given in the table: ns, not significant; *, P <0.05; **, P <0.01; and ***, P <0.001. Treatments: WW, irrigated daily to maintain 75-85% field capacity; DS, irrigated daily to maintain 45-55% field capacity; WW+DA, irrigated daily to maintain 75-85% field capacity plus 100 µM dopamine; and DS+DA, irrigated daily to maintain 45-55% field capacity plus 100 µM dopamine.

Element	WW	DS	WW+DA	DS+DA	Significances		
					DT	DA	DT×DA
N	25.22±0.18b	23.03±0.13d	25.69±0.15a	23.98±0.17c	***	***	**
P	1.78±0.04b	1.01±0.06d	2.25±0.05a	1.19±0.03c	***	***	***
K	17.00±0.19ab	15.25±0.42c	17.22±0.46a	16.49±0.25b	***	***	**
Ca	16.24±0.42b	22.28±1.15a	16.61±0.50b	17.42±0.47b	***	***	***
Mg	4.03±0.19b	4.96±0.57a	3.88±0.32b	4.03±0.23b	**	**	*
S	1.48±0.06a	1.40±0.05ab	1.45±0.05a	1.23±0.16b	**	*	ns
Fe	79.36±6.46a	80.45±3.35a	67.26±1.27b	71.07±6.97ab	ns	***	ns
Mn	36.55±0.34ab	40.67±2.79a	32.99±1.33b	35.46±3.35b	**	***	ns
Cu	5.31±0.23a	2.83±0.30c	5.63±0.29a	3.35±0.24b	***	**	ns
Zn	34.00±4.30a	28.19±2.62a	29.53±1.24a	32.80±3.43a	ns	ns	**
B	43.24±0.73a	33.96±1.49b	43.85±2.40a	33.13±1.11b	***	ns	ns
Al	53.71±4.88a	52.43±4.05a	40.12±4.61b	39.60±3.93b	ns	***	ns
Cr	0.37±0.05a	0.40±0.07a	0.32±0.04a	0.30±0.05a	ns	**	ns
Ni	0.84±0.11ab	0.98±0.03a	0.78±0.13b	0.91±0.08ab	**	ns	ns
As	1.19±0.08a	0.99±0.08bc	1.07±0.03ab	0.86±0.15c	***	*	ns
Mo	1.13±0.06b	0.63±0.03c	1.36±0.08a	0.72±0.08c	***	***	*
Pb	0.43±0.04ab	0.40±0.01b	0.37±0.02b	0.48±0.05a	*	ns	***
Cd	27.79±4.51a	31.24±1.84a	27.66±1.65a	25.84±3.56a	ns	ns	ns

TABLE S4 | Concentration of nutritional elements in stem dry matter from plants grown for 90 d under different watering and dopamine treatments. Data are means ± SD (n=5). Unit of measure: mg g^{-1} DW for N, P, K, Ca, Mg, and S; µg g^{-1} DW for Fe, Mn, Cu, Zn, B, Al, Cr, Ni, As, Mo, Pb, and Cd. An ANOVA test followed by Tukey's multiple range test was performed. Within a row, values not followed by the same letter indicate significant differences at $P_{0.05}$. level. Significant effects of the main factors drought (DT), dopamine (DA) and the interactions (DT×DA) are also given in the table: ns, not significant; *, P <0.05; **, P <0.01; and ***, P <0.001. Treatments: WW, irrigated daily to maintain 75-85% field capacity; DS, irrigated daily to maintain 45-55% field capacity; WW+DA, irrigated daily to maintain 75-85% field capacity plus 100 µM dopamine; and DS+DA, irrigated daily to maintain 45-55% field capacity plus 100 µM dopamine.

Element	WW	DS	WW+DA	DS+DA	Significances		
					DT	DA	DT×DA
N	12.05±0.12b	13.62±0.36a	12.03±0.13b	13.95±0.44a	***	ns	ns
P	1.06±0.05b	0.92±0.07c	1.24±0.08a	1.19±0.07a	**	***	ns
K	5.75±0.17bc	6.07±0.34ab	5.45±0.15c	6.21±0.11a	***	ns	*
Ca	6.29±0.56c	10.00±0.82a	6.12±0.12c	7.70±0.60b	***	***	**
Mg	1.35±0.09ab	1.48±0.05a	1.30±0.06b	1.26±0.09b	ns	**	*
S	0.39±0.04bc	0.62±0.02a	0.36±0.02c	0.44±0.02b	***	***	***
Fe	14.94±0.32ab	15.28±1.00a	12.76±1.43bc	11.01±1.10c	ns	***	ns
Mn	11.99±0.65a	8.36±0.44b	11.89±0.85a	8.11±0.67b	***	ns	ns
Cu	3.97±0.46ab	3.25±0.33c	4.57±0.44a	3.54±0.26bc	***	*	ns
Zn	9.74±0.43b	10.24±0.75b	9.43±0.85b	14.15±0.47a	***	***	***
B	60.90±0.45a	48.03±2.76b	60.14±1.23a	45.90±4.31b	***	ns	ns
Al	5.47±0.31a	4.59±0.31b	4.30±0.61b	5.39±0.45a	ns	ns	***
Cr	0.19±0.01a	0.12±0.02b	0.10±0.01b	0.10±0.01b	***	***	***
Ni	1.80±0.31a	0.94±0.10b	0.43±0.10c	0.76±0.11bc	**	***	***
As	0.16±0.02a	0.14±0.01ab	0.13±0.02ab	0.12±0.02b	*	*	ns
Mo	0.47±0.03a	0.27±0.01b	0.50±0.02a	0.30±0.03b	***	**	ns
Pb	0.12±0.02a	0.14±0.03a	0.05±0.01b	0.05±0.01b	ns	***	ns
Cd	15.60±1.75ab	19.81±2.95a	10.53±2.86c	15.35±1.56b	***	***	ns

TABLE S5 | Concentration of nutritional elements in root dry matter from plants grown for 90 d under different watering and dopamine treatments. Data are means ± SD (n=5). Unit of measure: mg g^{-1} DW for N, P, K, Ca, Mg, and S; µg g^{-1} DW for Fe, Mn, Cu, Zn, B, Al, Cr, Ni, As, Mo, Pb, and Cd. An ANOVA test followed by Tukey's multiple range test was performed. Within a row, values not followed by the same letter indicate significant differences at $P_{0.05}$. level. Significant effects of the main factors drought (DT), dopamine (DA) and the interactions (DT×DA) are also given in the table: ns, not significant; *, P <0.05; **, P <0.01; and ***, P <0.001. Treatments: WW, irrigated daily to maintain 75-85% field capacity; DS, irrigated daily to maintain 45-55% field capacity; WW+DA, irrigated daily to maintain 75-85% field capacity plus 100 µM dopamine; and DS+DA, irrigated daily to maintain 45-55% field capacity plus 100 µM dopamine.

Element	WW	DS	WW+DA	DS+DA	Significances		
					DT	DA	DT×DA
N	8.59±0.18d	15.98±0.25a	10.48±0.43c	15.40±0.29b	***	***	***
P	1.10±0.06b	0.88±0.10c	1.27±0.04a	1.04±0.08b	***	***	ns
K	4.58±0.10b	4.35±0.48c	5.42±0.11a	3.97±0.10bc	***	ns	***
Ca	7.88±0.18ab	8.71±0.75a	7.48±0.35b	5.93±0.50c	ns	***	***
Mg	1.48±0.11b	1.98±0.26a	1.47±0.06b	1.16±0.04c	ns	***	***
S	0.65±0.05b	1.08±0.19a	0.90±0.06a	0.65±0.04b	ns	ns	***
Fe	325.72±22.75c	628.89±105.87a	478.17±65.33b	418.93±56.29bc	**	ns	***
Mn	15.50±1.28b	25.85±5.83a	18.09±2.11b	13.62±0.95b	ns	**	***
Cu	7.52±0.38b	8.29±0.36ab	8.74±0.47a	5.73±0.59c	***	**	***
Zn	13.39±1.75a	9.43±0.94b	14.90±0.67a	6.70±0.90c	***	ns	**
B	60.90±0.45a	48.03±2.76b	60.14±1.23a	45.90±4.31b	***	ns	***
Al	437.25±80.71b	693.27±107.57a	510.65±57.69b	456.59±73.64b	*	ns	**
Cr	2.35±0.32b	3.72±0.75a	3.27±0.36a	2.96±0.19ab	*	ns	**
Ni	2.99±0.50a	3.27±1.16a	3.24±0.94a	2.25±0.43a	ns	ns	ns
As	0.84±0.08a	0.96±0.11a	0.84±0.18a	0.49±0.05b	*	***	***
Mo	1.05±0.14b	2.33±0.46a	1.44±0.04b	1.12±0.10b	***	**	***
Pb	0.46±0.03c	0.85±0.13a	0.65±0.03b	0.57±0.07bc	***	ns	***
Cd	72.39±6.66b	159.46±23.08a	87.31±13.54b	71.12±7.27b	***	***	***

TABLE S6 | Uptake fluxes of nutritional elements in plants after 90 d of growth under different watering and dopamine treatments. Data are means ± SD (n=5). Unit of measure: mg plant^{-1} day^{-1} for N, P, K, Ca, Mg, and S; μg plant^{-1} day^{-1} for Fe, Mn, Cu, Zn, B, Al, Cr, Ni, As, Mo, Pb, and Cd. An ANOVA test followed by Tukey's multiple range test was performed. Within a row, values not followed by the same letter indicate significant differences at $P_{0.05}$. level. Significant effects of the main factors drought (DT), dopamine (DA) and the interactions (DT×DA) are also given in the table: ns, not significant; *, P <0.05; **, P <0.01; and ***, P <0.001. Treatments: WW, irrigated daily to maintain 75-85% field capacity; DS, irrigated daily to maintain 45-55% field capacity; WW+DA, irrigated daily to maintain 75-85% field capacity plus 100 μM dopamine; and DS+DA, irrigated daily to maintain 45-55% field capacity plus 100 μM dopamine.

Element	WW	DS	WW+DA	DS+DA	Significances		
					DT	DA	DT×DA
N	18.68±0.09b	5.99±0.04d	21.68±0.33a	12.27±0.12c	***	***	***
P	1.78±0.07b	0.33±0.03d	2.27±0.06a	0.82±0.05c	***	***	ns
K	10.62±0.08b	2.44±0.12d	11.97±0.21a	4.90±0.08c	***	***	***
Ca	13.10±0.40b	4.14±0.16d	13.79±0.29a	6.15±0.16c	***	***	***
Mg	2.80±0.09a	0.87±0.05c	2.94±0.12a	1.24±0.03b	***	***	**
S	1.06±0.06b	0.37±0.04d	1.32±0.04a	0.52±0.01c	***	***	*
Fe	268.93±16.42b	136.54±21.98d	415.10±52.83a	197.00±25.12c	***	***	**
Mn	26.84±0.70b	8.83±1.28d	29.91±1.50a	11.89±0.28c	***	***	ns
Cu	8.80±0.33b	2.17±0.07d	10.93±0.54a	3.58±0.26c	***	***	*
Zn	23.57±1.37a	4.72±0.25c	25.14±0.80a	9.53±0.67b	***	***	**
B	79.04±1.70b	17.49±1.50d	92.52±1.78a	33.55±1.39c	***	***	ns
Al	340.00±58.75b	147.02±22.44c	429.72±48.56a	208.84±32.35c	***	**	ns
Cr	1.93±0.25b	0.80±0.16d	2.81±0.29a	1.37±0.08c	***	***	ns
Ni	3.25±0.42a	0.82±0.25b	3.08±0.80a	1.25±0.21b	***	ns	ns
As	1.02±0.05a	0.28±0.02b	1.06±0.15a	0.35±0.02b	***	ns	ns
Mo	1.30±0.10b	0.55±0.10c	1.80±0.04a	0.64±0.04c	***	***	***
Pb	0.51±0.03b	0.22±0.03d	0.66±0.03a	0.32±0.03c	***	***	ns
Cd	68.10±5.04b	36.65±4.52c	84.13±10.42a	37.51±3.05c	***	**	*

TABLE S7 | Transport of nutritional elements to leaves of plants grown for 90 d under different watering and dopamine treatments. Data are means ± SD (n=5). Unit of measure: μg plant^{-1} day^{-1} for N, P, K, Ca, Mg, and S; ng plant^{-1} day^{-1} for Fe, Mn, Cu, Zn, B, Al, Cr, Ni, As, Mo, Pb, and Cd. An ANOVA test followed by Tukey's multiple range test was performed. Within a row, values not followed by the same letter indicate significant differences at $P_{0.05}$ level. Significant effects of the main factors drought (DT), dopamine (DA) and the interactions (DT×DA) are also given in the table: ns, not significant; *, P <0.05; **, P <0.01; and ***, P <0.001. Treatments: WW, irrigated daily to maintain 75-85% field capacity; DS, irrigated daily to maintain 45-55% field capacity; WW+DA, irrigated daily to maintain 75-85% field capacity plus 100 μM dopamine; and DS+DA, irrigated daily to maintain 45-55% field capacity plus 100 μM dopamine.

Element	WW	DS	WW+DA	DS+DA	Significances		
					DT	DA	DT×DA
N	104.61±1.18a	27.09±0.62c	106.87±0.97a	42.70±0.79b	***	***	***
P	7.54±0.23b	-0.89±0.30d	10.52±0.30a	0.84±0.15c	***	***	***
K	83.20±1.28a	33.96±2.03c	83.62±2.96a	44.36±1.17b	***	***	***
Ca	72.85±1.69a	61.88±1.34b	72.38±0.60a	42.69±0.90c	***	***	***
Mg	17.63±0.36a	11.79±2.05b	17.64±1.25a	8.27±0.33c	***	*	*
S	6.31±0.29a	2.27±0.12b	5.93±0.08a	2.37±0.47b	***	ns	ns
Fe	374.47±19.87a	147.36±7.02c	260.81±6.09b	140.15±4.11c	***	***	***
Mn	185.07±1.69a	129.01±3.94c	163.17±6.43b	101.91±11.14d	***	***	ns
Cu	28.79±0.35a	5.29±0.69c	29.71±1.17a	7.95±0.18b	***	**	ns
Zn	214.98±0.12a	107.32±3.14d	175.91±3.59b	141.62±5.71c	***	ns	***
B	190.35±1.85b	38.38±1.51d	201.01±6.16a	50.66±2.23c	***	***	ns
Al	215.12±2.65a	79.43±7.41c	132.12±14.58b	51.19±12.61d	***	***	**
Cr	-0.51±0.08a	-1.99±0.10c	-0.46±0.12a	-1.64±0.16b	***	*	ns
Ni	2.37±0.46a	0.40±0.08b	2.36±0.44a	0.92±0.25b	***	ns	ns
As	6.67±0.17a	3.45±0.09c	5.72±0.09b	3.17±0.16c	***	***	**
Mo	5.37±0.20b	-0.23±0.08d	6.30±0.22a	0.96±0.06c	***	***	ns
Pb	2.64±0.09a	1.38±0.04c	1.95±0.11b	1.79±0.17b	***	ns	***
Cd	167.62±10.13a	97.71±3.44c	139.48±8.39b	68.29±10.67d	***	***	ns

TABLE S8 | Transport of nutritional elements to stems of plants grown for 90 d under different watering and dopamine treatments. Data are means ± SD (n=5). Unit of measure: μg $plant^{-1}$ day^{-1} for N, P, K, Ca, Mg, and S; ng $plant^{-1}$ day^{-1} for Fe, Mn, Cu, Zn, B, Al, Cr, Ni, As, Mo, Pb, and Cd. An ANOVA test followed by Tukey's multiple range test was performed. Within a row, values not followed by the same letter indicate significant differences at $P_{0.05}$ level. Significant effects of the main factors drought (DT), dopamine (DA) and the interactions (DT×DA) are also given in the table: ns, not significant; *, P <0.05; **, P <0.01; and ***, P <0.001. Treatments: WW, irrigated daily to maintain 75-85% field capacity; DS, irrigated daily to maintain 45-55% field capacity; WW+DA, irrigated daily to maintain 75-85% field capacity plus 100 μM dopamine; and DS+DA, irrigated daily to maintain 45-55% field capacity plus 100 μM dopamine.

Element	WW	DS	WW+DA	DS+DA	Significances		
					DT	DA	DT×DA
N	109.73±1.27a	44.77±2.02c	108.04±1.32a	65.49±2.84b	***	***	***
P	9.86±0.57b	2.63±0.38d	11.59±0.91a	5.73±0.46c	***	***	*
K	52.80±1.83a	19.57±1.90d	48.86±1.57b	28.82±0.71c	***	**	***
Ca	53.16±3.27a	34.50±3.65b	51.55±1.03a	32.79±3.27b	***	ns	ns
Mg	11.67±0.39a	4.24±0.19c	11.54±0.38a	5.38±0.35b	***	*	*
S	3.57±0.29a	2.39±0.05b	3.15±0.14a	1.97±0.09b	***	**	ns
Fe	46.03±3.13a	-77.81±2.59c	28.43±12.04b	-61.61±2.68c	***	ns	**
Mn	112.27±0.75a	27.01±1.07c	110.90±7.14a	37.55±2.05b	***	ns	*
Cu	38.19±4.35b	9.46±0.40d	46.15±3.25a	17.02±0.77c	***	**	ns
Zn	92.65±1.05a	32.45±1.05c	90.23±6.03a	74.08±1.88b	***	***	***
B	621.12±1.36a	194.86±5.91d	595.22±12.95b	259.95±12.21c	***	**	***
Al	36.10±2.45a	-6.14±0.76d	20.04±3.40b	7.86±0.29c	***	ns	***
Cr	0.69±0.03a	-1.25±0.03d	-0.38±0.11b	-0.94±0.05c	***	***	***
Ni	-0.97±0.58a	-20.07±0.27d	-13.91±0.63b	-15.54±0.37c	***	***	***
As	1.56±0.08a	0.49±0.01b	1.31±0.20a	0.53±0.02b	***	ns	*
Mo	4.46±0.13a	0.60±0.01c	4.72±0.25a	1.36±0.11b	***	***	*
Pb	1.03±0.10a	0.34±0.09b	0.25±0.05b	-0.08±0.07c	***	***	**
Cd	111.87±9.80a	25.23±5.03c	68.21±7.21b	26.74±5.98c	***	***	***

TABLE S9 | Accumulation of nutritional elements in roots of plants grown for 90 d under different watering and dopamine treatments. Data are means ± SD (n=5). Unit of measure: μg plant^{-1} day^{-1} for N, P, K, Ca, Mg, and S; ng plant^{-1} day^{-1} for Fe, Mn, Cu, Zn, B, Al, Cr, Ni, As, Mo, Pb, and Cd. An ANOVA test followed by Tukey's multiple range test was performed. Within a row, values not followed by the same letter indicate significant differences at $P_{0.05}$ level. Significant effects of the main factors drought (DT), dopamine (DA) and the interactions (DT×DA) are also given in the table: ns, not significant; *, P <0.05; **, P <0.01; and ***, P <0.001. Treatments: WW, irrigated daily to maintain 75-85% field capacity; DS, irrigated daily to maintain 45-55% field capacity; WW+DA, irrigated daily to maintain 75-85% field capacity plus 100 μM dopamine; and DS+DA, irrigated daily to maintain 45-55% field capacity plus 100 μM dopamine.

Element	WW	DS	WW+DA	DS+DA	Significances		
					DT	DA	DT×DA
N	70.38±3.29c	115.95±3.61b	112.44±7.90b	163.15±4.84a	***	***	ns
P	12.95±1.12b	3.55±1.37d	16.86±0.65a	9.81±1.34c	***	***	*
K	50.63±1.74b	19.91±6.80d	69.46±2.01a	31.08±1.72c	***	***	*
Ca	92.70±1.63a	58.92±3.69b	87.01±1.10a	44.20±4.16c	***	***	*
Mg	16.58±0.41a	10.62±1.08b	16.87±0.80a	7.27±0.01c	***	**	**
S	4.88±0.57b	7.96±1.29a	9.51±0.70a	3.15±0.35b	**	ns	***
Fe	930.54±121.13b	1248.03±380.89b	3544.40±104.96a	1437.55±221.35b	*	**	**
Mn	157.73±11.61b	237.94±48.79a	218.67±26.12ab	77.26±2.93c	ns	*	***
Cu	76.42±5.23b	40.91±0.89c	108.54±5.79a	36.86±3.77c	***	***	***
Zn	145.21±3.31b	35.61±5.26c	199.64±10.55a	39.83±9.60c	***	***	***
B	754.83±22.95b	512.39±25.86c	949.21±8.39a	569.5±27.18c	***	***	***
Al	2396.69±623.91b	2679.72±550.96ab	3996.04±234.63a	1791.77±703.62b	*	ns	**
Cr	-10.52±1.49b	-13.06±2.46b	15.18±5.64a	-5.37±2.23b	***	***	**
Ni	-22.78±3.13a	-47.17±6.11b	-22.39±14.10a	-50.50±1.92b	***	ns	ns
As	7.41±0.54ab	4.85±1.06bc	9.91±3.22a	0.86±0.15c	***	ns	*
Mo	4.65±0.24b	15.01±3.14a	14.32±0.47a	4.13±0.80b	ns	ns	***
Pb	-0.81±0.06b	1.63±1.31a	3.25±0.09a	-0.84±0.73b	ns	ns	***
Cd	-1783.79±77.75ab	-1830.66±233.32b	-1457.60±111.97a	-2215.97±68.79c	**	ns	**

TABLE S10 | Nutrient resorption proficiency for elements in leaves from plants grown for 120 d under different watering and dopamine treatments. Data are means ± SD (n=5). Unit of measure: mg g^{-1} DW for N, P, K, Ca, Mg, and S; μg g^{-1} DW for Fe, Mn, Cu, Zn, B, Al, Cr, Ni, As, Mo, Pb, and Cd. An ANOVA test followed by Tukey's multiple range test was performed. Within a row, values not followed by the same letter indicate significant differences at $P_{0.05}$. level. Significant effects of the main factors drought (DT), dopamine (DA) and the interactions (DT×DA) are also given in the table: ns, not significant; *, P <0.05; **, P <0.01; and ***, P <0.001. Treatments: WW, irrigated daily to maintain 75-85% field capacity; DS, irrigated daily to maintain 45-55% field capacity; WW+DA, irrigated daily to maintain 75-85% field capacity plus 100 μM dopamine; and DS+DA, irrigated daily to maintain 45-55% field capacity plus 100 μM dopamine.

Element	WW	DS	WW+DA	DS+DA	Significances		
					DT	DA	DT×DA
N	9.39±0.16d	11.65±0.25b	11.07±0.14c	12.69±0.10a	***	***	***
P	0.80±0.01b	0.41±0.02d	1.07±0.07a	0.50±0.01c	***	***	***
K	10.34±0.17d	10.78±0.26c	13.08±0.14a	11.53±0.21b	***	***	***
Ca	27.00±0.33a	21.40±1.17c	20.52±0.53c	23.24±0.64b	***	***	***
Mg	3.75±0.09b	3.81±0.29ab	3.64±0.05b	4.11±0.05a	**	ns	*
S	0.80±0.07ab	0.70±0.06b	0.86±0.07a	0.83±0.04a	*	**	ns
Fe	116.14±7.77a	69.86±6.68c	89.31±4.36b	97.25±3.41b	***	ns	***
Mn	16.16±1.99c	24.63±3.56ab	20.79±1.28bc	26.23±2.26a	***	*	ns
Cu	3.63±0.36b	2.03±0.08c	3.90±0.26a	2.73±0.25c	***	**	ns
Zn	30.79±3.38a	17.20±0.34c	31.71±2.91a	25.79±2.54b	***	**	**
B	28.18±2.68a	21.99±0.73b	31.63±2.52a	28.91±2.43a	***	***	ns
Al	102.98±6.02a	56.37±7.18c	79.58±3.84b	82.33±7.39b	***	ns	***
Cr	0.50±0.03a	0.35±0.05b	0.48±0.07a	0.41±0.04ab	***	ns	ns
Ni	0.54±0.06ab	0.49±0.04b	0.54±0.06b	0.65±0.07a	ns	**	**
As	1.26±0.05a	1.01±0.10b	1.13±0.06ab	1.08±0.06b	***	ns	**
Mo	0.39±0.04b	0.35±0.03b	0.48±0.06a	0.39±0.04b	**	**	ns
Pb	0.66±0.04a	0.43±0.03c	0.65±0.03a	0.53±0.01b	***	**	***
Cd	49.11±3.61a	33.26±2.84b	45.98±4.51a	36.71±2.63b	***	ns	ns

TABLE S11 | Concentration of nutritional elements in leaf, stem, and root dry matter from plants grown for 0 d under different watering and dopamine treatments. Data are means ± SD (n=5). Unit of measure: mg g^{-1} DW for N, P, K, Ca, Mg, and S; μg g^{-1} DW for Fe, Mn, Cu, Zn, B, Al, Cr, Ni, As, Mo, Pb, and Cd.

Element	Leaf	Stem	Root
N	29.29±0.15	13.10±0.21	13.32±0.14
P	2.00±0.12	1.06±0.06	1.07±0.11
K	13.79±0.20	6.00±0.18	5.01±0.11
Ca	16.56±0.42	8.94±0.71	7.85±0.69
Mg	4.14±0.34	1.67±0.07	1.78±0.12
S	1.62±0.09	0.46±0.03	1.09±0.11
Fe	84.38±2.25	68.51±4.97	800.24±60.73
Mn	26.62±1.30	8.43±0.51	21.63±0.86
Cu	2.94±0.38	3.11±0.48	9.28±0.43
Zn	9.58±0.39	9.36±0.48	11.64±0.76
B	45.50±1.28	30.33±0.57	33.16±1.73
Al	56.01±1.81	13.94±0.72	807.91±43.00
Cr	1.30±0.16	0.81±0.06	7.85±0.64
Ni	1.52±0.16	10.75±0.31	12.51±0.68
As	0.55±0.04	0.12±0.02	1.08±0.12
Mo	1.11±0.07	0.37±0.04	2.05±0.17
Pb	0.19±0.02	0.18±0.02	1.38±0.10
Cd	17.60±0.42	40.98±4.71	487.56±35.30
